# Effect of crown-to-implant ratio and crown height space on marginal bone stress: a finite element analysis

**DOI:** 10.1186/s40729-021-00368-1

**Published:** 2021-09-01

**Authors:** José Joaquim da Rocha Ferreira, Luís Filipe Meira Machado, José Manuel Oliveira, João Carlos Tomás Ramos

**Affiliations:** 1 Institute of Science and Innovation in Mechanical and Industrial Engineering (INEGI), Oporto, Portugal; 2 Centre of Mathematics, University of Minho, Campus de Azurém, 4800-058 Guimarães, Portugal; 3 Department of Dentistry, Stomatology and Maxillofacial surgery, Faculty of Medicine, University of Coimbra, Av. Bissaya Barreto-Blocos de Celas, 3000-075 Coimbra, Portugal

## Abstract

**Background:**

Crown-to-implant ratio and crown height space, associated with the use of short implants, have been related with marginal bone loss. However, it is unclear which of the two entities would play the most important role on the bone remodelling process. Using a finite element analysis, the present work aims to help clarifying how those two factors contribute for the stress generation at the marginal bone level. A numerical model (reference model), with a crown-to-implant ratio of 4, was double validated and submitted to a numerical calculation. Then, it was modified in two different ways: (a) by decreasing the prosthetic height obtaining crown-to-implant ratios of 3, 2.5 and 2 and (b) by increasing the implants length obtaining a crown-to-implant ratio of 2.08. The new models were also submitted to numerical calculations.

**Results:**

The reference model showed a marginal bone stress of 96.9 MPa. The increase in the implants’ length did not show statistically significant differences in the marginal bone stress (p-value = 0.2364). The decrease in the prosthetic height was accompanied with a statistically significant decrease in the marginal bone stresses (p-value = 2.2e− 16).

**Conclusions:**

The results represent a paradigm change as the crown height space appears to be more responsible for marginal bone stress than the high crown-to-implant ratios or the implants’ length. New prosthetic designs should be attempted to decrease the stress generated at the marginal bone level.

## Background

Although the oral rehabilitation with dental implants is a predictable and well-documented technique [1–3], long-term survival may be hampered by biological, technical, physiological and biomechanical factors [4–6].

Among the biomechanical factors, the crown-to-implant ratio (CIR) has been object of research. It is defined as the relationship between the crown and the implant lengths. If the crown length is measured from the implant platform an anatomical CIR is defined. Instead, if the bone level is taken into account to measure the crown length, it is defined as a clinical CIR [7, 8].

The research on this concept is associated with the use of short implants, since their use frequently result in prosthetic rehabilitations with high crowns and, therefore, the creation of a potentially deleterious CIR.

The rationale behind the concern with the CIR is related with the bending effect as a consequence of the horizontal components of mastication loads. These off-centre forces generate bending stresses which maximum values are located at the implant-abutment connection and at the marginal bone area, with potentially deleterious consequences on implants and prosthetic connections, screws and marginal bone [9, 10]. The concern with the CIR also results from the adoption of the tooth supported fixed prosthesis similar concept, where the minimal crown-to-root ratio recommended is 1:1 and, ideally, the crown should be smaller than the supporting root [11]. As the dental implants were equated to the teeth roots, the initial implant dentistry advised the limit of a 1:1 CIR, similar to the crown-to-root ratio [7].

After an initial period, when the emerging short implants were associated with lower survival rates [12–15], some aspects contributed to establish their safety of use [16–18] and consider them as a viable alternative to more complex surgical approaches, when the available bone volume is scarce [19]. Among those aspects are the implant macro design changes, the surface treatments, the development of new implant-abutment connections, the introduction of the platform shifting concept and new surgical protocols [20]. Interventions on the prosthesis were also proposed including the implants ferulization [21], the reduction of the mesio-distal and lingual-buccal cantilevers [22] or directing the forces to a more axial direction to reduce the lever created during mastication [23].

Therefore, the use of short implants in maxillary and mandible posterior areas is showing an exponential increase, creating new fields of investigation, with particular focus on their biomechanical performance [24–26]. The major concern is to understand the limits of their use, namely, to what extent the CIR might be a safe clinical option. A consensus report on biomechanics and risk management [27] stated that “the use of implant-supported restorations with C/I ratios up to two do not influence crestal bone loss”. Malchiodi et al. [28], in a prospective study, suggested that the critical threshold value to avoid bone loss and implant failure would be an anatomical CIR of 3.10 and a clinical CIR of 3.4.

However, different conclusions were published. Lee et al. [29], Pazmino et al. [30] and Rokni et al. [31] found that the higher the CIR, the smaller the marginal bone loss, reporting an inverse relationship between both variables.

A photoelastic study [32] and a finite element analysis work [33] found a correlation between an increased CIR and higher marginal bone stress levels only when oblique loads were applied.

Blanes [8], Okada et al. [34] and Esfahrood et al. [35] stated that the CIR does not influence the marginal bone maintenance. A longitudinal cohort study conducted by Ramaglia et al. [36] suggested that bone loss is neither related to implant length nor to anatomical CIR. An in vitro experiment published by Nissan et al. [37] reported that a different factor, the crown height space (CHS), would be more significant than CIR in assessing biomechanical-related detrimental effects, recommending vertical bone augmentation procedures when the CHS is 15 mm or longer [38]. The same author advised that the studies assessing the effects of CIR should also mention CHS in their results. The absence of that reference may explain the different correlations reported by different authors between the CIR and the marginal bone loss [39].

The CHS was defined as the distance from the crest of the bone to the plane of occlusion [40]. This entity affects the reactions at the marginal bone level, once it dictates the lever arm mechanism in the presence of lateral components of the biting forces [41, 42]. A greater crown height results in a greater bending moment which increases the stresses in the implant-bone interface [7]. Even if, due to the pressure regulated nature of the bone metabolism [43, 44], within the microphysiological range, this may induce a stimulation of bone formation [29], if the individual bone’s elastic limit is overcome it may result in bone remodelling [45]. In fact, Misch affirmed that each millimetre of CHS increase is expected to result in a 20% augmentation of the reaction forces at the marginal bone level [41].

Anitua et al. [46] did not find a statistical correlation between the CIR and the crestal bone loss. However, the author could find a positive significant correlation between the crestal bone loss and the CHS. The biomechanical explanation behind this finding is based on the fact that the marginal bone is the most responsible for load support [47], reason why the higher level of stress is found at that region, regardless the implant length. An increase in the implant length would not be effective to counterbalance the prosthetic height effect [46].

In fact, finite element analysis (FEA) have shown that the reactions to occlusal forces are concentrated in the crestal bone and not throughout the entire bone-to-implant interface, despite the implant length [48, 49]. This simulation method, used to predict the biomechanical performance of a system, is a qualitative and approximation method which contributes to a better understanding of a biomechanical problem [9]. This tool has been widely used in dental implantology to evaluate the biomechanical behaviour of living tissues and restorative materials [50, 51]. It allows the multiplication of tests in a small period of time, varying the critical factors of each experience, in a way that it would be undoable with experimental biomechanics, animal or clinical testing. However, the results are highly dependent on calculation conditions. Its accuracy is extremely dependent on factors such as geometry, material and tissue properties, boundary and loading conditions, type of element, mesh sensitivity and contact definitions [24, 52].

Thus, because differences will always exist between the virtual model and reality, to rely on the FEA results, an experimental validation is required [53, 54], before the conduction of clinical trials.

The justification of the present work is to predict the stress state in the marginal bone around short dental implants for different CIR and CHS conditions. Therefore, aiming to identify which of those factors is of most importance for stress generation at the marginal bone level.

## Methods

This research work was divided in five steps: (a) geometry and numerical modelling, (b) finite element analysis, (c) model validation, (d) analysis of the model’s behaviour under different CIR and (e) statistical analysis.

**Fig. 1:**
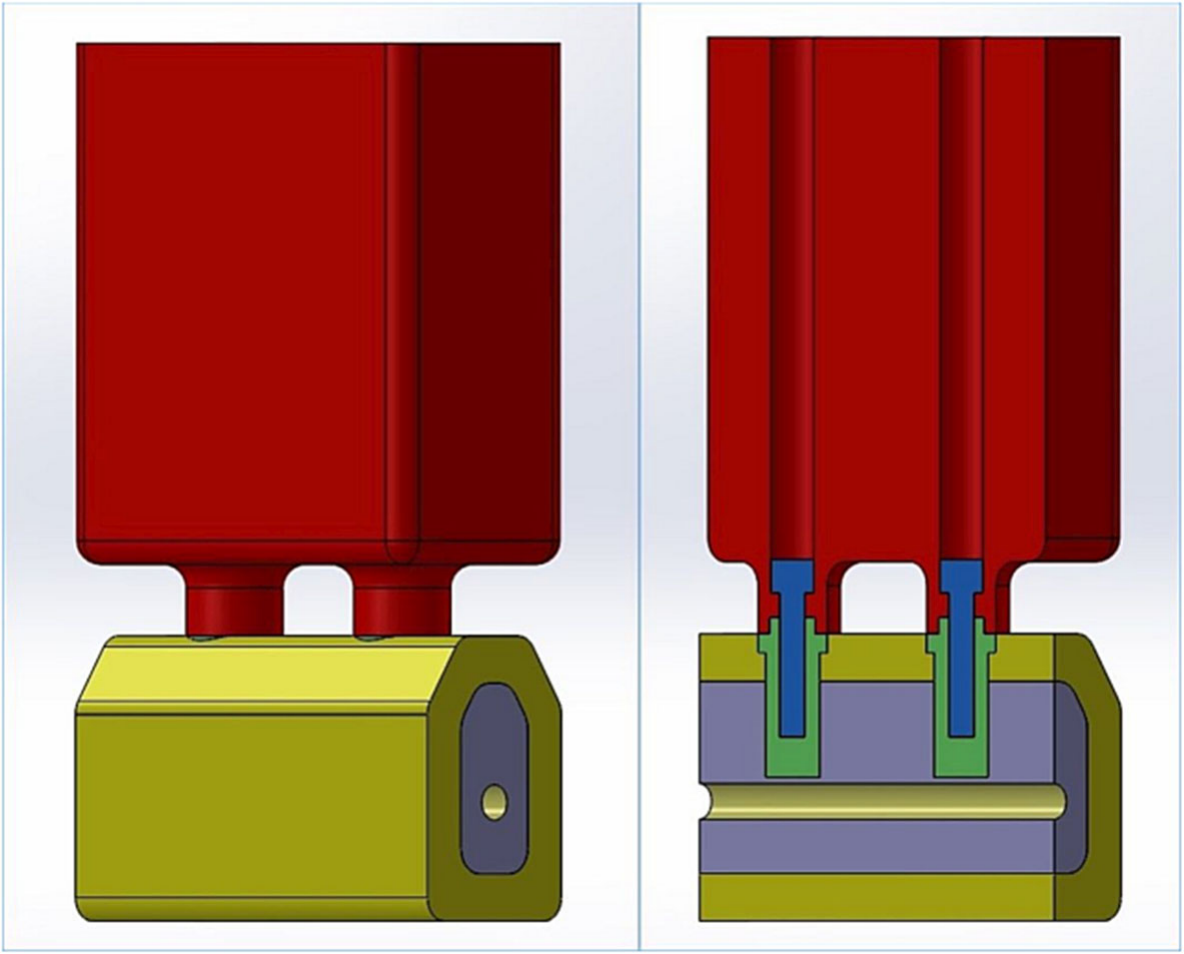
Geometrical details of the study model. On the left: external cut-view of the model assembly. On the right: mesio-distal cut-view of the model exhibiting the internal aspects of the assembly: cortical bone (yellow), trabecular bone (purple), implants (green), implant screws (blue) and prosthetic framework (red). It is also possible to observe the implant screw channels and the mandibular canal

A simplified geometry design, simulating a posterior mandibular rehabilitation, was obtained using the SolidWorks™ software (Dassault Systemes SA, Velizy, France) (Fig. 1). The model consisted of the following parts:

1. *Bone*. The measures were obtained from patients’ computerized tomographies, allowing for the design of two implants supporting a partial implant fixed prosthesis with 2 teeth (first and second molars). The bone was divided in two different parts: a cortical part, exterior, with a thickness of 2 mm [55], to simulate a type II bone, and a trabecular part, constituted by the internal portion.
2. *Dental endosseous implants and prosthetic screws*. Simplified geometries were modelled with recourse to product technical data and reverse engineering. To test the worst case scenario [56, 57], external hexagon short implants (4.1 mm wide and 6 mm long), were chosen. The implant platform was placed at the marginal bone level.
3. *Prosthetic framework*. Modelled considering the approximate dimensions of 2 molars. The height of 24 mm was calculated by the difference between the model total height, which resulted from computerized tomography measurements, and the bone height, thus obtaining anatomical CIR of 4, exceeding the safe CIR proposed by Malchiodi et al. [28].

The numerical model was obtained by transferring the geometrical model to the Abaqus™ software (Dassault Systemes SA, Velizy, France). Elastic materials’ properties were assigned to each model part [58, 59] regarding the transversely isotropy of the bone [60] (Table 1) and considering the other materials as isotropic (Table 2). All the materials were considered solid and homogeneous. Interactions [61] and boundary conditions were defined (Table 3).

**Table 1:** Elastic properties adopted for the bone

	Young’s modulus, MPa			Shear modulus, MPa			Poisson coefficient		
	E_x_	E_y_	E_z_	G_xy_	G_yz_	G_xz_	ν_xy_	ν_yz_	ν_xz_
Cortical bone	12600	12600	19400	4850	5700	5700	0.3	0.253	0.253
Trabecular bone	1148	210	1148	68	68	434	0.055	0.01	0.322

**Table 2:** Materials and elastic properties defined for implants, implant screws and prosthetic framework

	Young’s modulus (MPa)	Poisson coefficient
Prosthetic framework (cobalt-chrome alloy)	194 000	0.30
Implants (grade IV titanium alloy)	105 000	0.37
Implant screws (grade V titanium alloy)	120 000	0.33

**Table 3:** Interactions and boundary conditions elected for the present study

Friction coefficients	Co-Cr with Ti gr IV (Prosthetic framework with implant platform)	0.15
	Co-Cr with Ti gr V (Prosthetic framework with screw’s head)	0.15
	Ti gr V with Ti gr IV (Screw’s body with inner implant platform)	0.43
Tie constrain	Screw’s body with inner implant body (simulating the threaded connection)	
Merge	Outer implant’s surface with cortical and trabecular bone (simulating osteointegration)	
Encastre	As boundary condition on the lower face of the bone (preventing rotations and/or displacements)	

**Fig. 2:**
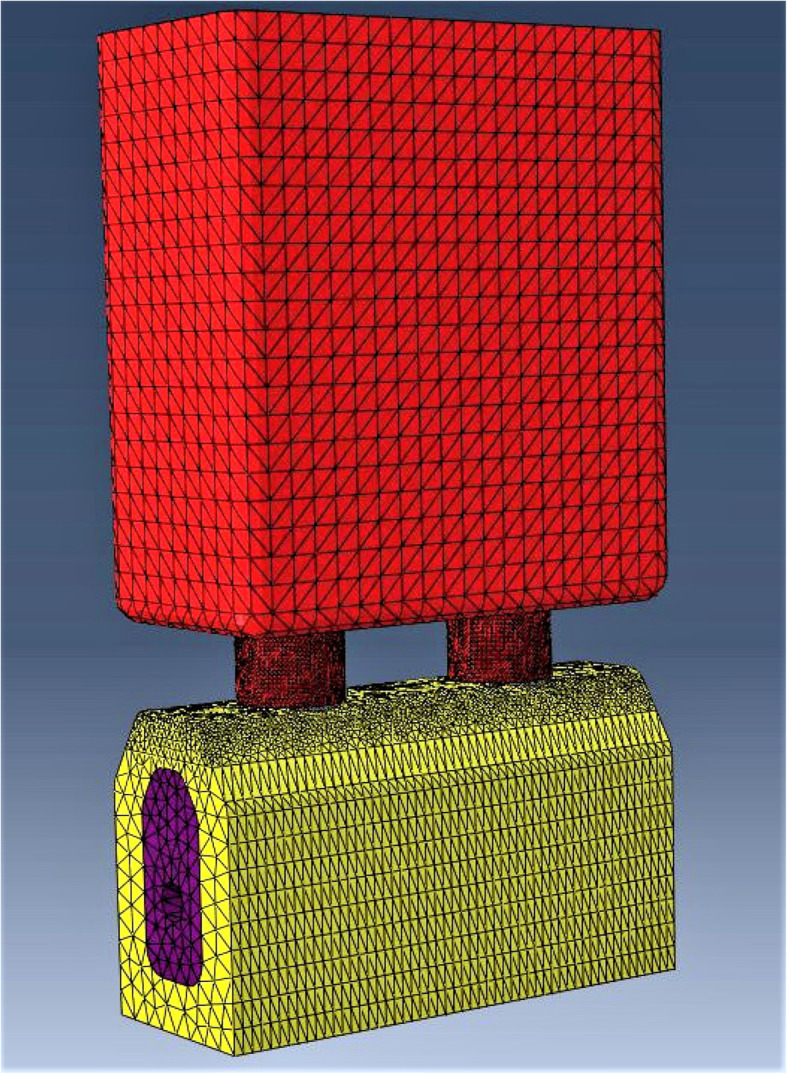
Numerical model discretization where an area with a refined mesh at the marginal bone and prosthetic connectors can be observed

A mesh sensitivity analysis was performed with two goals: to achieve the stress convergence to obtain a mesh size that would allow for consistent predictions in the shortest simulating time possible [62] and to identify the areas where major stresses would be computed, enabling a refined mesh in those locations and a coarse mesh elsewhere (Fig. 2). The element chosen was tridimensional, tetrahedral and second order (C3D10) which permitted the discretization of all the geometries present in the model [63]. With these definitions, an automatic discretization generated the elements and nodes shown in Table 4.

**Table 4:** Number of nodes and elements (C3D10) obtained for each model part

	Number of nodes	Number of elements
Bone	116 821	77 868
Implants (each)	24 518	16 146
Prosthetic framework	205 335	140 338
Implant screws (each)	1 003	550

The loading scenario consisted in a force with a magnitude of 23 N and a 30° slope, directed from lingual to buccal, distributed to 3 circular areas of 0.63 mm^2^ simulating the occlusal contacts, located at the buccal third of the occlusal face, which resulted in a pressure of 36.54 N [64].

An implicit static simulation [65, 66] with one step for the load appliance, was conducted with the described numerical model (for identification purposes named as “reference model”).

The results were validated with a double approach: a quasi-static compressive load test and an electronic speckle pattern interferometry (ESPI) test. Both tests intend to compare the mechanical behaviour of the numerical model with the experimental model. The former confirmed the sequence of failure of the models parts [67] and the last confirmed the displacement behaviour [68].

After model validation, several iterations were carried out in the CIR. It was changed in two different manners: (a) in a first set of experiments, the prosthetic framework height was decreased to 18 mm (CIR = 3), 15 mm (CIR = 2.5) and 12 mm (CIR = 2) maintaining the original short implants; (b) in a second experiment, the implants were changed to 11.5 mm length maintaining the prosthetic framework initial height, which resulted in a CIR of 2.08. With this methodology, it was possible to analyse the evolution of the computed stresses in the marginal bone when the CIR decreases as a consequence of a lower CHS and as a result of the use of longer implants. It was, as well, possible to compare both methods of reducing the CIR and understand which factor would be of most importance on the stress levels generation in the marginal implant bone.

**Fig. 3:**
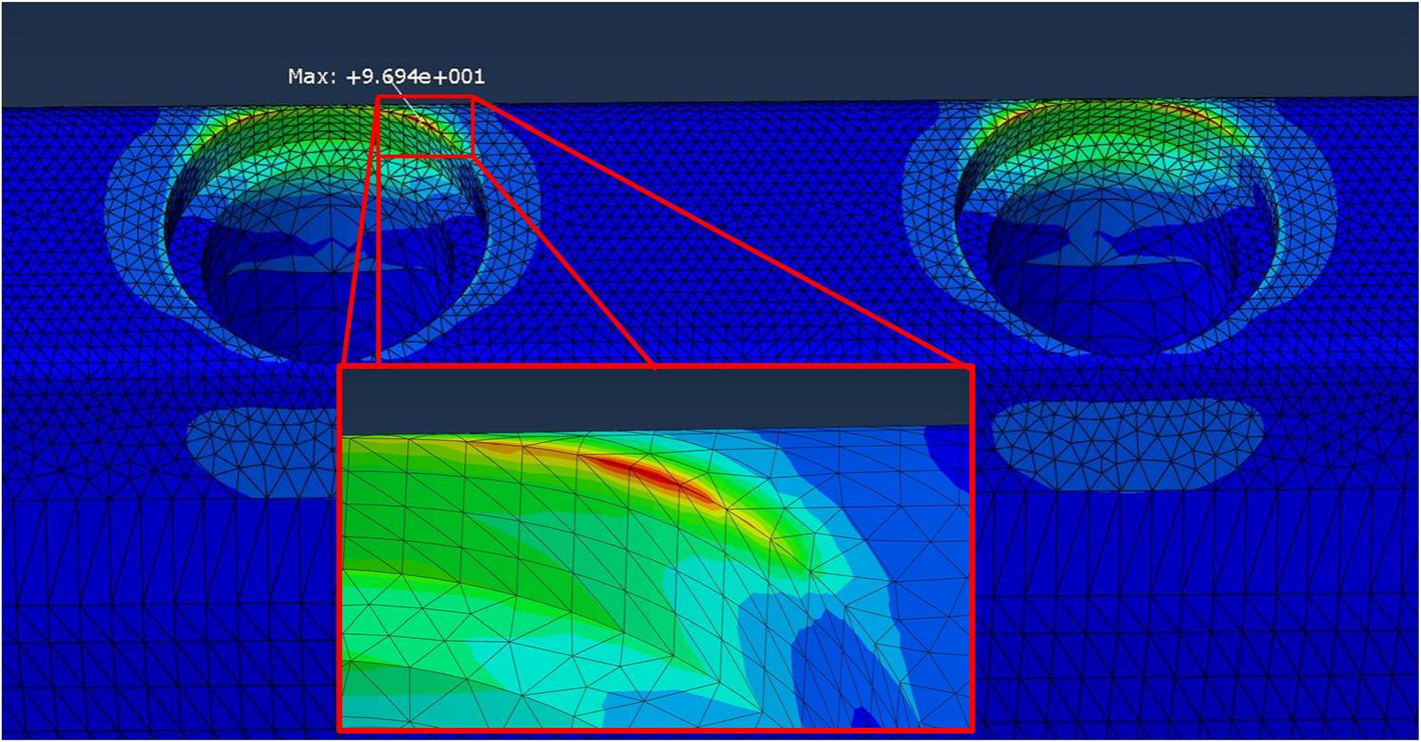
Location of the areas where the highest stresses were predicted in the bone part and magnification of one of those four areas of interest. Location and value of the highest stress computed for the bone part (96.94 Mpa)

Due to the numerical nature of these types of work, additional series of observations would have the same results. So, some FEA do not present statistical analysis [69, 70]. However, for each model, it is possible to select the same area of interest, where the higher von Mises equivalent stresses are found, and calculate a median stress value, which could be compared between the different models [71]. Thus, for each bone part, four areas of interest were considered, as illustrated in Fig. 3. Different information was collected from those areas: (1) the highest computed stress (Fig. 3), (2) the stress computed for each element node and (3) the average stress predicted for each element centre.

The non-parametric Wilcoxon rank-sum test was chosen to compare the central tendency between the different models. The null hypothesis, for each comparison, is that there are no differences, in the stress values, between the models. For all hypotheses testing, an α = 0.05 significance level was used.

## Results

### Reference model

**Fig. 4:**
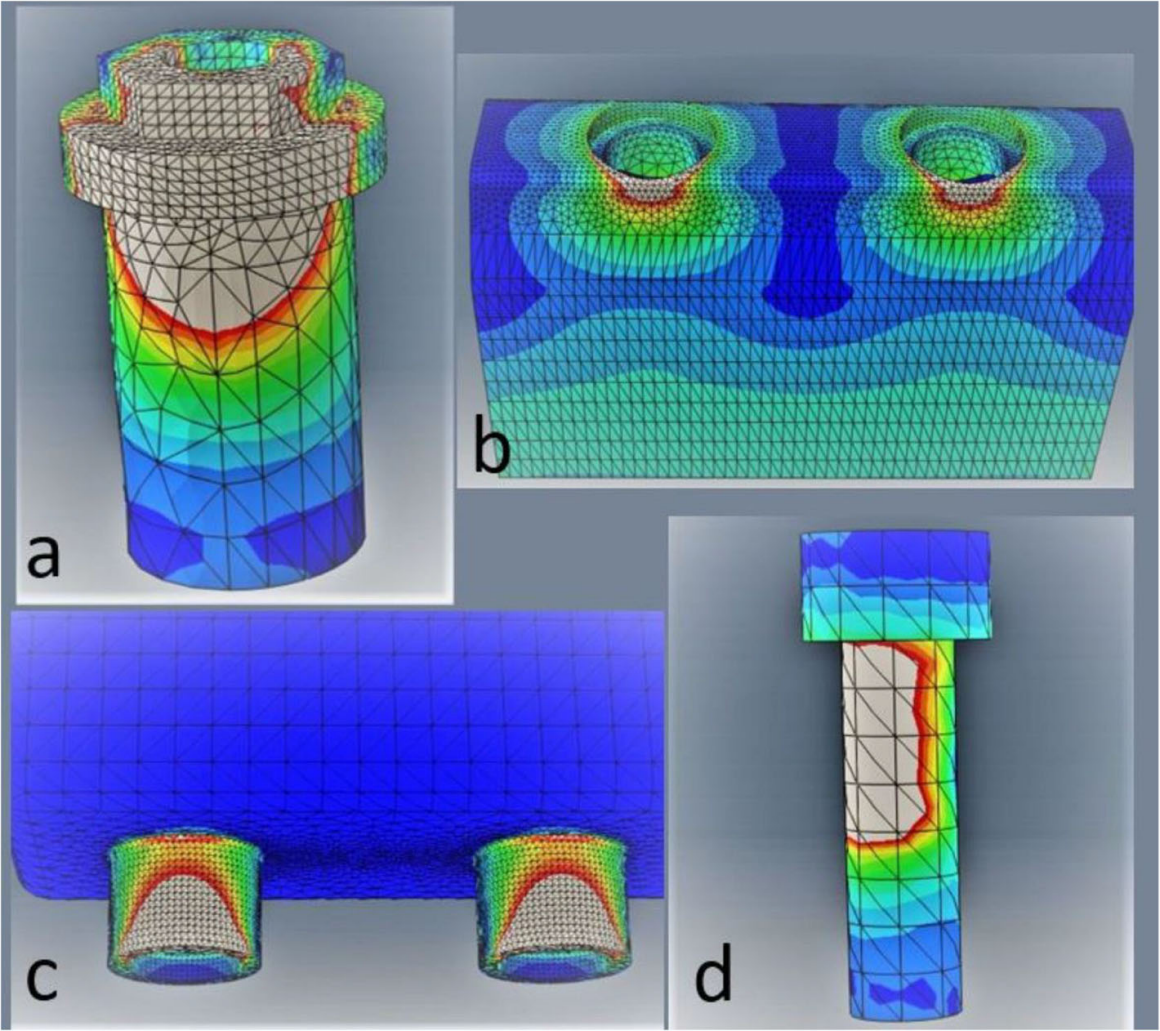
Stress pattern in the reference model: **a** Short implants. The grey areas represent stresses above 60 MPa. **b** Bone part. The grey areas present stresses above 30 MPa. **c** Prosthetic framework. The grey areas represent stresses above 150 MPa. **d** Prosthetic screws. The grey regions present stresses above 200 MPa

The stress computed for the reference model is exposed quantitatively in Table 5 and qualitatively in Fig. 4. The quantitative results show that the bone is the only material that exceeds the yield strength, considering Frost’s mechanostat theory [45]. The remaining materials present an adequate factor of safety for clinical use and would be unlikely to deform permanently with the physiologic applied load conditions. For this reason, and because it is the only biological tissue considered in this study, for the purpose of discussing the results and their clinical interpretation, bone will be the only part of the model to be analysed.

**Table 5:** Highest stress values according to the von Mises criterion for each reference model part and its relation with the materials’ yield strength

FEA				SCLT
	von Mises stress (MPa)	Materials’ yield strength (MPa)	Factor of safety	Sequence of failure/deformation
Bone	96.9	60	0.62	First
Prosthetic framework	326.9	659	2.02	Second
Implants	351.6	750	2.13	Third
Implant screws	344.6	795	2.30	Fourth

The factor of safety is presented in the third column (ratio of the maximum stress that a material can withstand to the maximum stress it will be subjected to in function). Relation with the SCLT

Observing the bone areas described above, a median stress of 58.74 MPa (SD = 17.6) was obtained when considering the element nodes and a median stress of 59.57 MPa (SD = 4.8) when considering the centre of each element.

Regarding the qualitative results, the area where the major stresses are expected in the bone is the marginal bone in the buccal surface of the implant platform.

### Reference model validation

As this is a basic science research, only an experimental biomechanical approach was considered.

The model validation was obtained by confirming a similitude of tendencies or behaviours more than an absolute correspondence of the values measured. It is not a quantitative comparison but rather an evidence of a qualitative equivalence. For this purpose, the bone part was materialized with an epoxy resin with a Young’s modulus of 20 MPa, a Poisson coefficient of 0.363 and a yield strength of 45 MPa. These mechanical characteristics were obtained by static tensile load tests according to the standards ASTM D-638-02a [72] and ISO 527-1 [73]. These properties were assumed in the numerical model.

#### Quasi-static compressive loading test (SCLT)

**Fig. 5:**
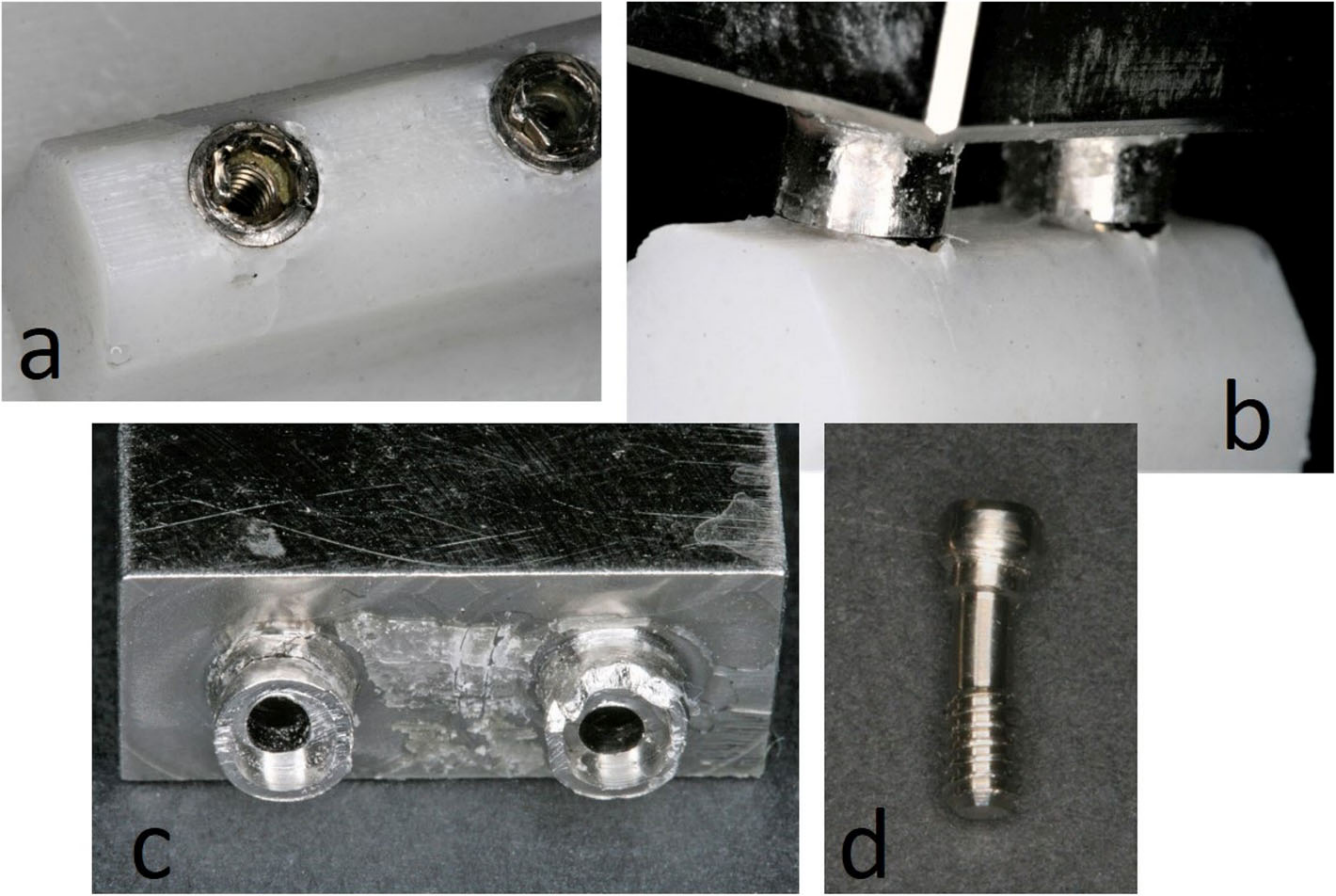
Damage in the experimental model: **a** Fractures verified in the vestibular face of the implants’ hexagons, as predicted after resign failure (compare with Fig. 4a). **b** Damage in the resin that simulates the bone tissue. Comparing with **b**, it is possible to visualize that similar zones are affected. **c** Damage observed in the prosthetic framework connector, in accordance with the simulation results (compare with **c**). **d** Slight screw deformation found after the finished SCLT, similar to the results presented in **d**

This test allowed to establish a positive correlation between the sequence of failure on the experimental model and the evolution of stresses predicted in the numerical model. The sequence was identical for both models indicating that the first part to deform or fracture would be the resin simulating bone, followed by the prosthetic framework, the implants and finally the screws (Table 5). The SCLT also shows that the fractures/deformations, for each model component, occurred in the locations where higher stresses were predicted by the numerical model (Figs. 4 and 5).

#### Electronic speckle pattern interferometry (ESPI)

**Fig. 6:**
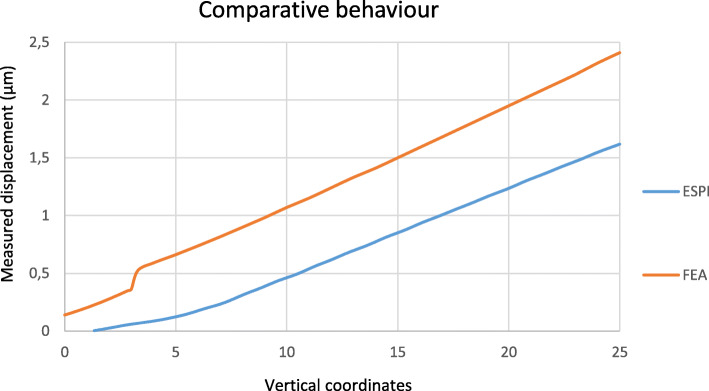
Comparison between the displacements of the FEA (orange) and the average of the samples used during the ESPI test

**Fig. 7:**
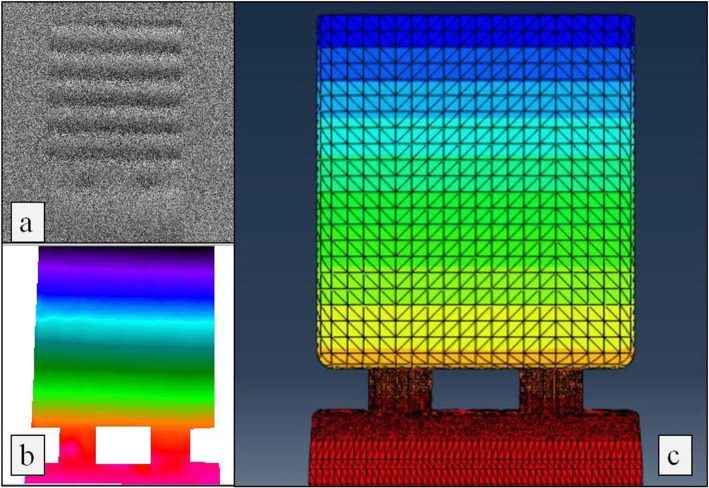
Coincidence of displacements calculated with ESPI and FEA. The ESPI results may be visually translated by a fringe pattern that represents the displacement field of the surface under study: **a** Its transduction into a colour code (**b**) results in an image similar to the obtained on FEA (**c**), facilitating the comparison between both models’ results

A qualitative analysis of the results showed that both, the numerical model and the tested samples, exhibited identical linear behaviour, with the resulting displacement being consistent with a rotation. Both indicate the geometric difference between the prosthetic connectors and the body of the prosthetic framework. To allow a direct comparison with the numerical model, an arithmetic average of the displacements measured in the experimental samples was calculated and formatted for similar loads and vertical coordinates. The result showed a parallelism in the behaviour of both models (Figs. 6 and 7).

### Model’s behaviour under different CIR

**Fig. 8:**
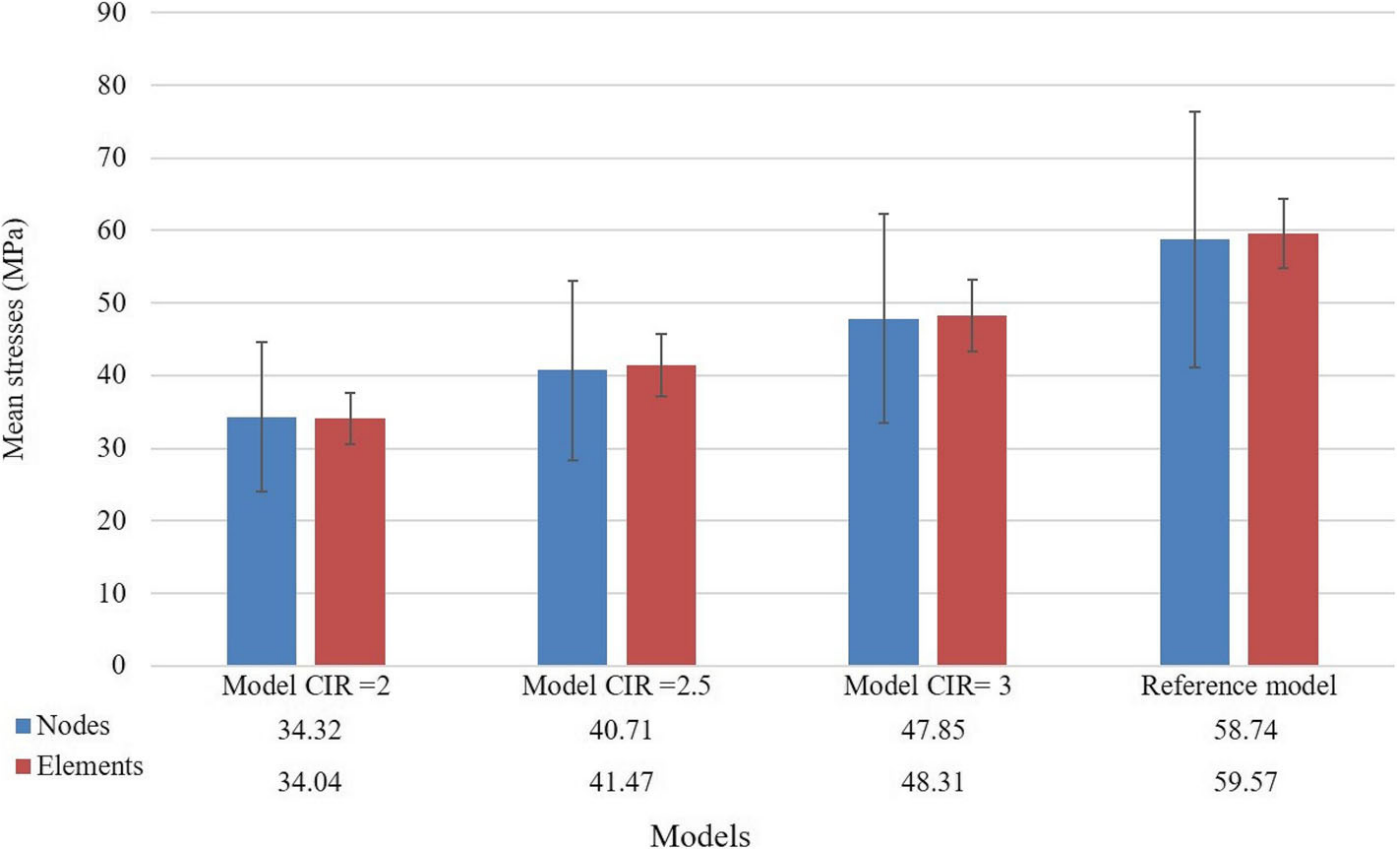
Evolution of the median stresses predicted in the areas of interest with the change in the prosthetic height. It is possible to observe that the increase in the CIR and in the prosthetic height corresponds to an increase in the stress predicted

**Fig. 9:**
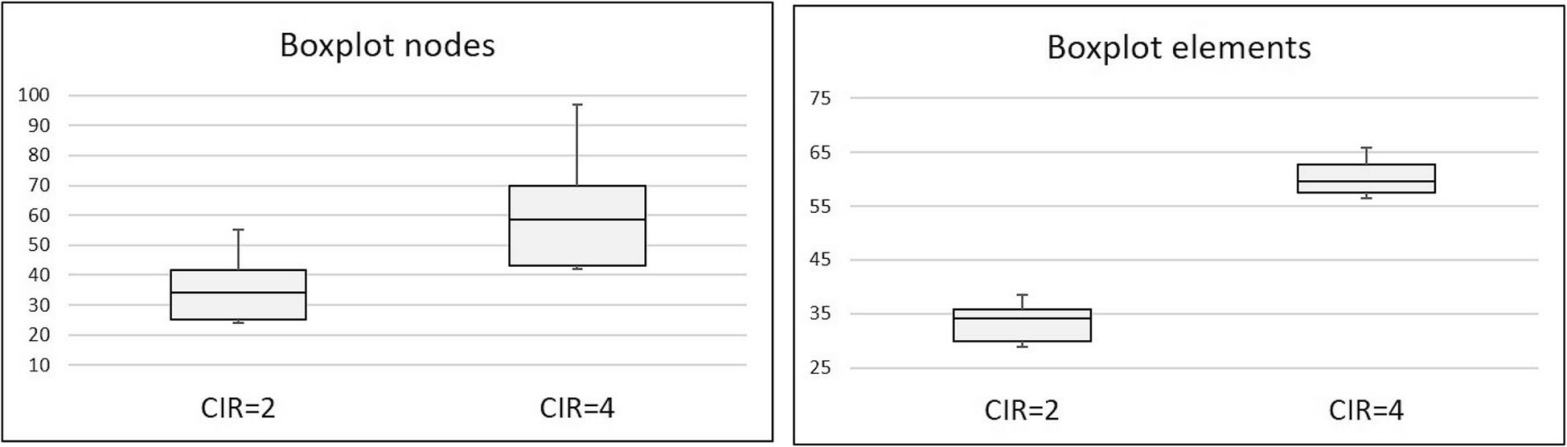
Comparison between the central tendencies of the reference model and the CIR = 2 model, putting in evidence the significant differences found

When the CIR was modified by decreasing the prosthetic framework height, the stresses computed in the implant bone margin also decreased. The results of the highest stress, the median stress measured in the nodes and the median stress measured in the elements’ centre are depicted in Table 6 and Fig. 8. This set of calculations maintained the implants dimensions. It was possible to observe that the stress predicted in other model components, such as implants, screws and prosthesis, was under the yield strength of the elected materials. A highest stress prediction under the yield strength considered for the bone (60 MPa) is achieved somewhere between a CIR of 2 and 2.5, for a prosthetic height between 12 and 15 mm. The statistical analysis comparing the reference model with the CIR = 2 model showed that both models exhibit statistically significant differences, allowing the rejection of the null hypothesis (p-value = 2.2e− 16 for the median stress of the nodes and p-value = 1.079e− 14 for the median stress in the elements’ centre) (Fig. 9).

**Table 6:** Evolution of the stresses (MPa) predicted in the FEA when the CIR decreased by decreasing the prosthetic height and maintaining of the implants’ length

	CIR = 4, reference model	CIR = 3	CIR = 2.5	CIR = 2
Bone	96.9	79.2	69.8	55.1
Implants	351.6	315.3	272.9	289.2
Implant screws	344.6	237.9	204.9	181.1
Prosthetic framework	326.9	254.3	215.4	174.6

When the CIR was modified by increasing the implants length to 11.5 mm, keeping the prosthetic height (CIR = 2.08), it was observed that the major stress predicted for the marginal bone is higher than the bone’s yield strength, while the other model parts are in a safe range (Table 7).

**Table 7:** Stress (MPa) prediction for the model with a CIR of 2.08 obtained by increasing the implants’ length. A comparison with the reference model and with the model with a similar CIR may also be observed

	CIR = 4 (reference model)	CIR = 2.08 (11.5-mm implants)	CIR = 2 (short implants)
Bone	96.9	101.6	55.1
Implants	351.6	442.5	289.2
Implant screws	344.6	308.4	181.1
Prosthetic framework	326.9	347.3	174.6

**Fig. 10:**
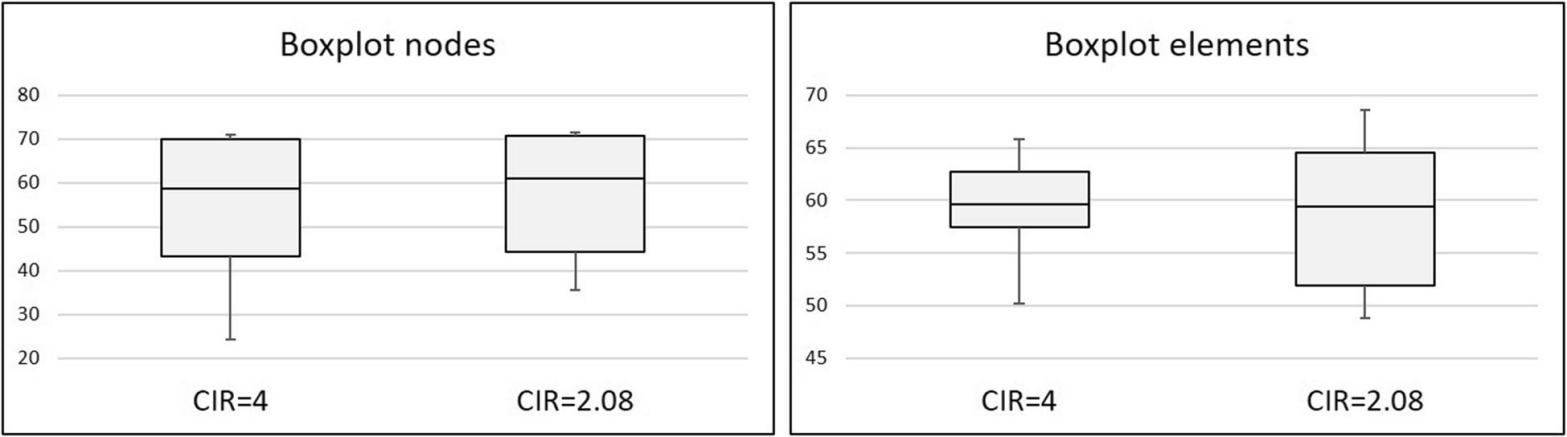
Boxplot comparing the reference model with the CIR = 2.08 model highlighting the absence of significant differences between the stresses found in both models

**Fig. 11:**
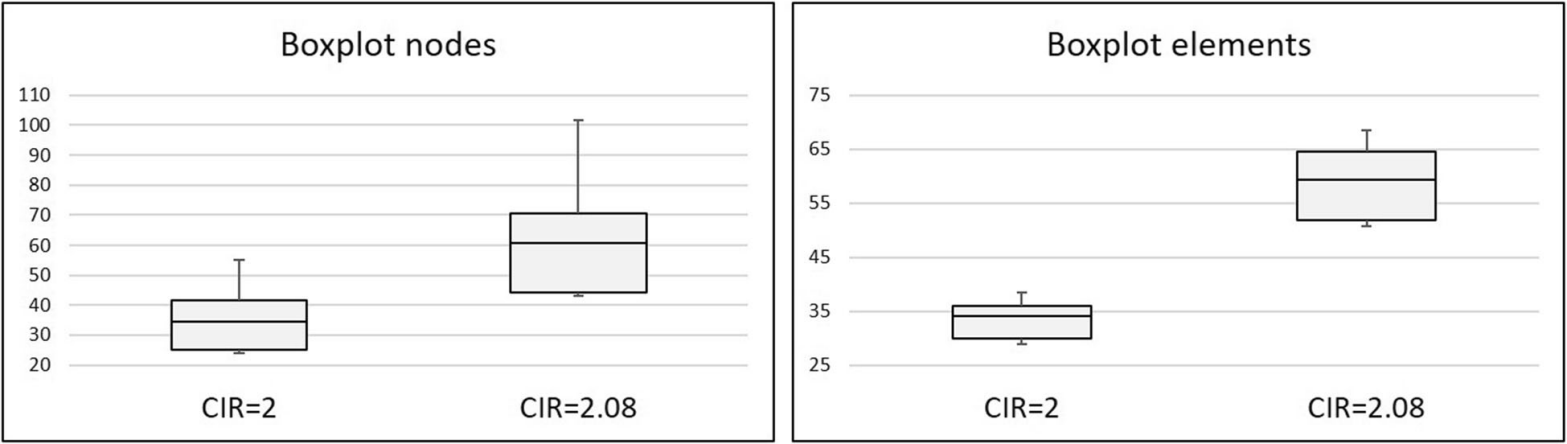
Boxplots showing the significant differences shown by the comparison between the stresses computed in the CIR = 2 model and the CIR = 2.08 model

Analysing the same bone areas, a median stress of 60.92 MPa (SD = 18.6) was obtained when considering the elements’ nodes and a median stress of 59.43 MPa (SD = 7.1) when considering the centre of each element. These results may be analysed in two different manners:

1. *Comparing them with the reference model (CIR = 4)*. Both models have the same prosthetic height but different implant lengths. Both showed the highest bone stress value higher than the bone’s elastic limit. The Wilcoxon rank-sum test was used to compare the node stresses. A p-value of 0.2364 was obtained which led to the conclusion that, for a level of significance of 5%, there are no statistically significant differences in the stress values given by the two models, confirming the null hypothesis. The same conclusion may be obtained when considering the stresses in the elements’ centre (p-value = 0.8297) (Fig. 10).
2. *Comparing them with the model CIR = 2*. Despite having similar CIR (2.08 and 2), both models have different prosthetic heights and different implant lengths. Comparing the central tendencies when considering the node stresses, the Wilcoxon rank-sum test, for a level of significance of 5%, obtained a p-value inferior to 2.2e− 16, which allows the rejection of the null hypothesis: both models exhibit statistically significant differences which means that the model with short implants, but also shorter prosthetic height exhibits the best results. As well, the same conclusion is achieved when comparing the stresses at the elements’ centre (p-value = 3.535e− 09) (Fig. 11).

## Discussion

The reference model simulates a rehabilitation of the posterior mandible, where the option was the placement of short implants at the expense of opting for vertical bone regeneration procedures. As result, short implants were combined with a high CHS resulting in a high CIR with potential detrimental effects.

Under the tested conditions, and taking into account the necessary caution in the interpretation of finite element studies, which results cannot be freely extrapolated to a clinical scenario, the computed stresses, confronted with Frost’s theory [45], are placed in an interval compatible with bone remodelling. This theory considered that the bone’s ability to spontaneously heal is surpassed when the stress reaches approximately 60 MPa. Because the calculation of the bone’s elastic properties is difficult, some different studies indicate different elastic limits for the cortical bone. Maximum values of 140 MPa, 121 MPa or 117 MPa [74] were proposed by different authors. However, even considering those different elastic limits proposed, the predicted von Mises equivalent stress values for the marginal bone around the implants’ buccal faces, still place this material as the most likely to reach the yield strength. A similar result was found by Himmlová et al. [24] who found that the maximum stress areas were located around the implant neck. Nissan et al. [38] also stated that non-axial forces, as the mastication and the load scenario of the present study, are concentrated at the crest of the implant supporting bone. A FEA published by Sutpideler et al. [10] also predicted that the highest stress concentrations would be found in the cortical bone surrounding the implant platform.

The relation of the marginal bone stress with the CIR and CHS was investigated by the modifications performed in the reference model. The first modification seems to indicate that a decrease in the CIR results in a lower von Mises equivalent stress levels in the marginal bone. The bone’s elastic limit is exceeded for a CIR between 2.5 and 2. Apparently, these results are in agreement with the consensus that stated the safety of a CIR up to two [27] and contradict the Malchiodi et al. [28] threshold of a 3.1 CIR. However, a more careful reading of the results should take into account that the CIR decrease is only due to the prosthetic height decrease. This fact could result in a biassed conclusion that the second modification in the reference model intended to clarify.

This experiment used a model with a CIR close to 2, maintaining the prosthetic height and increasing the implants’ length from 6 to 11.5 mm. However, no statistically significant differences were found in the bone stresses exhibited by both models, indicating that the modification in the implants’ length had no positive impact on the marginal bone stress computed. Therefore, the identical results could not be due neither to different CIR nor to different implant lengths, pointing to the identical CHS as the explanation for the similar results. As well, comparing this model’s results with the model with identical CIR (= 2) but a smaller CHS (12 mm), it shows a von Mises predicted stress in a safe zone (55.1 MPa) while the former, with a similar CIR (= 2.08) and a higher CHS (24 mm) predicts a bone stress that overcomes the yield strength proposed by Frost (101.6 MPa). Both models showed statistically significant differences in the bone stresses computed. So similar CIR could not be responsible for statistically significant differences.

These results seem to indicate that the CHS may be more important for the implant therapy success than the CIR or the implants’ length. This is aligned with the studies from Anitua et al. [46] that found a positive correlation between the CHS and the crestal bone loss, together with an absence of compensation by an increase in the implant length. It is also in line with the studies from Sutpideler et al. [10] who found that the stresses were increased when the height of the prostheses increased, regardless of the angle of the applied force or the amount of offset. A study from Ramaglia et al. [36] had already suggested that the marginal bone loss was neither influenced by the implants’ length nor by the CIR.

**Fig. 12:**
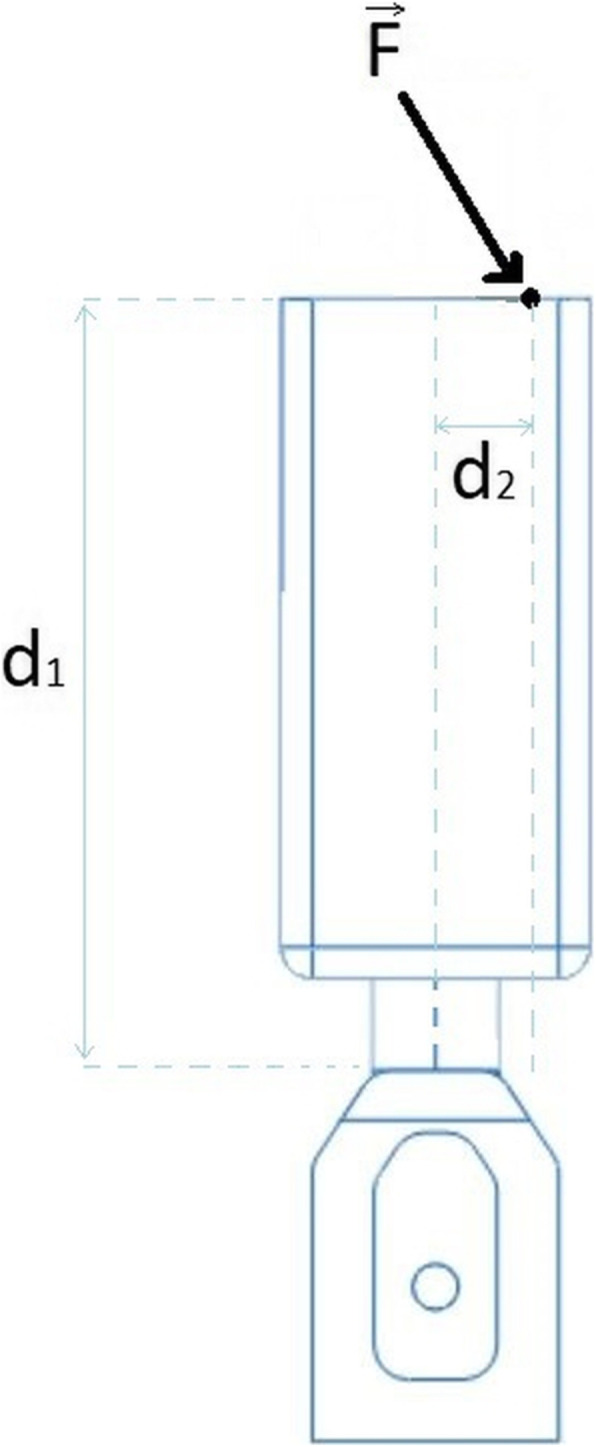
Schematic diagram of the reference model with the representation of the applied force and the distances d1 and d2 responsible for the bending moments on the bone and, therefore, the generation of high stresses at the margin bone level

This behaviour was expected having in consideration the static equilibrium of the system (Fig. 12) which could be defined by the following expressions regarding the reactions at the bone level:

1a $$ \sum \overrightarrow{F}=\overrightarrow{0} $$

and

1b $$ \sum \overrightarrow{T}=\overrightarrow{0} $$

2a $$ \sum \overrightarrow{F}=\overrightarrow{F}+\overrightarrow{R}=\overrightarrow{0} $$

and

2b $$ \sum \overrightarrow{T}=\overrightarrow{T}+\overrightarrow{TR}=\overrightarrow{0} $$

$\overrightarrow{F}$represents the applied force.

$\overrightarrow{R}$represents the reaction at the bone margin at the implant-abutment connection level.

$\overrightarrow{T}$and$\overrightarrow{TR}$are respectively the torque resultant of the applied force and the reaction torque.

$\overrightarrow{F}$ and $\overrightarrow{T}$ are respectively given by the expressions:

3a $$ \overrightarrow{F}=-\overrightarrow{R}=\left\{\begin{array}{c} Fx\\ {} Fy\\ {} Fz\end{array}\right. $$

and

3b $$ \overrightarrow{T}=-\overrightarrow{TR}=\left\{\begin{array}{c} Tx\\ {} Ty\\ {} Tz\end{array}\right. $$

Because the applied load (F) does not have a component on the ZZ axis, Fx is the horizontal component of the load, Fy is the vertical component and Fz is equal to zero. For the same reason, there is no torque (T) defined for the XX and YY axes but there is a torque on the ZZ axis due to the bending effect. This torque is given by the following equation:

4a $$ \overrightarrow{F}=\left\{\begin{array}{c} Fh\\ {} Fv\\ {}0\end{array}\right. $$

and

4b $$ \overrightarrow{T}=\left\{\begin{array}{c}0\\ {}0\\ {}\left( Fh\times d1\right)+\left( Fv\times d2\right)\end{array}\right. $$

These expressions show that, if the applied load and the load application point are maintained constant, although the reaction forces will remain the same, the torque reactions decrease as d1 decreases, due to its importance on the torque calculation. Considering that the stress could be understood as the internal reaction forces divided per unit area, it is expectable that decreasing the prosthetic height, the stress generated at the bone crest would also decrease.

Due to the exposed results and corroborating the publications of Nissan et al. [37, 38], the studies assessing the effects of CIR should mention both implant length and CHS in their results. The possible problems may begin for a CHS around 15 mm, as it happened in this study. This might be the reason why publications such as those of Blanes [8], Okada et al. [34] and Esfahrood et al. [35] did not find a relation between the CIR and bone loss while other authors like Lee et al. [29] or Pazmino et al. [30] reported an inverse relationship between both entities and Sotto Maior et al. [32] or Moraes et al. [33] found a correlation between the CIR and the prediction of a higher stress levels in the margin bone. None of these studies analysed the CHS.

This represents a paradigm change. Papers and clinicians focus their attention on the implant length, but the results exposed indicate that the attention should be put on the prosthesis height. This way, if the CHS is smaller than 15 mm, the results suggest that no bone augmentation procedures are needed and a minimally invasive, less time consuming, cheaper and simpler rehabilitation with recourse to short implants may be an efficient procedure. It is suggested, by these results, that the good performance showed by the rehabilitations based on vertical bone augmentation procedures do not result from the placement of longer implants but from a lower prosthetic height.

New prosthetic designs could be object of research, considering the clinical cases where the CHS is higher than 15 mm, to decrease to levels below the yield strength the stress generated in the implant marginal bone.

### Study limitations

Certainly, the discussion of the present study does not exclude the multifactorial aetiology of bone response to implant rehabilitation. Among others, factors such as the implant’s diameter, micro and macro geometry, implant-abutment connection, bone’s quality and volume, surgical protocols or the passive fit of the prosthetic components, should be also taken into account.

One possible limitation of the present study is the use of simplified geometries. Nevertheless, simplification reduces the possibility of interaction conflicts and shortens the simulation time. More simulations, with the same CHS and different implant lengths, would also increase the consistency of the results. As well, the parameters chosen for the FEA, such as the vertical implant position, the implant-abutment connection, the implant’s cervical geometry, the absence of an intermediate abutment and the loading scheme, have an impact in the FEA results. Different choices could have achieved different results. However, all the assumptions made on the numerical model definition, had the intention of, at the same time, simplifying the model and resembling clinical situations.

Likewise, different materials could be tested for either the prosthetic framework, screws, or implants, which may lead to different results. In fact, only two more different materials were considered for the prosthetic framework: zirconia and the titanium grade V alloy. The safety factors for those materials were, respectively, of 2.66 and 2.93, generating von Mises stresses at the cortical bone of 97.1 MPa (safety factor, 0.62) and 94.1 MPa (safety factor, 0.64). Comparing these results with those obtained with the cobalt-chrome alloy, it is possible to identify a similar safety factor for the stresses computed for the cortical bone, but the framework exhibits the lowest safety factor of the three materials tested, reason why it was elected for the study.

The results of this study indicate that the prosthetic screw loose is unlikely to occur, a fact that contradicts the clinical practice. This apparent contradiction may be explained because the chosen loading model, although with parameters such as value, direction and application areas, which attempted to mirror average physiological bite parameters, corresponds to a single mastication cycle. A sequence of cycles would be better simulated with a fatigue test, which was not performed in this research context. It also should be taken into account that loose screws may not always be the first occurrence and may have already associated some marginal bone loss or other factors not considered in this study, namely a poor implant-abutment fit or incorrect tightening torque.

## Conclusions

Within the limitations of this research, a high crown height space appears to be the most responsible factor for the marginal bone stress generation. It seems that a paradigm change may emerge, where the prosthetic height is more important than the implants’ length or the ratio between the protheses’ height and the implants’ length. In this scenario, research on this topic is needed to develop new prosthetic designs that could decrease the stress generated at the marginal bone level.

Knowing that the presence of greater stresses in the marginal bone may be related to a greater probability of initiating a remodelling process, this finding is clinically relevant once it may guide the clinician during the elaboration of a treatment planning, expanding the limits of a rehabilitation with short implants which is a simple, conservative, less invasive and less expensive technique. This finding also opens the possibility for the clinicians that are not comfortable with more complex surgical techniques to perform a simpler and safer rehabilitation. However, more studies are necessary, including clinical randomized controlled trials, to strengthen the conclusions of this research and the option for this restorative approach.

## Data Availability

The datasets used and/or analysed during the current study are available from the corresponding author on reasonable request.

## Abbreviations

CIR: Crown-to-implant ratio
CHS: Crown height space
FEA: Finite element analysis
TM: Trademark symbol
ESPI: Electronic speckle pattern interferometry
SD: Standard deviation
ASTM: American society for testing and materials
ISO: International organization for standardization
SCLT: Quasi-static compressive loading test
